# Knowledge, Attitude, and Practice of Nursing Home Staff Regarding Physical Restraint in China: A Cross-Sectional Multicenter Study

**DOI:** 10.3389/fpubh.2022.815964

**Published:** 2022-05-06

**Authors:** Yaqin Li, YaWen Wang, Yechun Gu, Daqiang Gong, Sisi Jiang, Jufang Li, Hongbo Xu

**Affiliations:** ^1^School of Nursing, Wenzhou Medical University, Wenzhou, China; ^2^General Surgery Department, Wenzhou Hospital of Integrated Traditional Chinese and Western Medicine, Wenzhou, China; ^3^Zhejiang Dongfang Polytechnic, Wenzhou, China

**Keywords:** physical restraint, nursing staff, knowledge, attitude, practice

## Abstract

**Background:**

Restraint is widely used in nursing homes to address safety concerns. However, many studies have shown that improper restraint can lead to many adverse outcomes. Nursing staff are the main practitioners of physical restraint in nursing homes and play an important role in restraint decision-making and management. In China, there is still a lack of large-scale surveys on the current situation regarding the use of restraint. This study aimed to identify this situation and the influencing factors of the knowledge, attitude, and practice of nursing staff regarding physical restraint in elderly care institutions.

**Methods:**

A cross-sectional multicenter descriptive study was conducted. A total of 311 staff in 25 elderly care institutions in Zhejiang Province were recruited using a quota sampling method. A homemade questionnaire was administered to collect general information and information on knowledge, attitude and practice regarding physical restraint.

**Results:**

The average scoring rates of the knowledge and attitude dimensions of the 311 staff were 48.7 and 75.6%, respectively. The average scoring rate of the practice dimension of 140 staff who implemented restraint was 80.1%. Educational background and training experience were the influencing factors of restraint knowledge. Training experience, educational background and professional title were the influencing factors of restraint attitude. Restraint knowledge and length of service were the influencing factors of restraint practice.

**Conclusions:**

Knowledge of physical restraint among nursing staff is not promising. Their attitude toward restraint was inappropriate or irresponsible in some aspects. Restraint practice is not sufficiently standardized. It is necessary to strengthen restraint training for nursing staff to improve their overall level of restraint knowledge, attitude and practice.

## Introduction

Physical restraint refers to the use of physical or mechanical devices, materials, or tools attached to or adjacent to the patient's body that cannot be easily removed, thus limiting the patient's activities or making the patient unable to normally move his or her body (1). Currently, physical restraint is used mainly in medical institutions such as intensive care units, medicine wards and surgery wards (2), especially in neurosurgery, geriatrics, and psychiatry departments (3). The main purpose is to prevent accidental withdrawal of the patient's tubes and violent injuries.

With the growing aging population and changing attitudes toward elderly care, there will be an increasing proportion of elderly people choosing to live in elderly care institutions. In China, the number of elderly people in nursing homes is relatively high (4). Among elderly individuals, physical function and self-care ability gradually worsen with age (5). At the same time, a variety of chronic illnesses and medications also increase the risk of injury to older people through falls and other accidents (6). Furthermore, a previous study showed that the more frequent the physically aggressive behavior of elderly individuals, the more likely they are to be constrained (7). To reduce the occurrence of accidents and protect the safety of both elderly individuals and others, the use of restraint can be applied under certain circumstances in elderly care facilities (8–10). A study conducted in China illustrated that the prevalence of physical restraint was 25.83% (11).

However, some studies have shown that improper use of restraint can not only fail to achieve the purpose of protection but also have many adverse effects on the physical and mental health of elderly individuals, such as urinary incontinence, asphyxia, falls, limb dysfunction, aggravation of cognitive impairment, increased dependence on others, decline in daily life abilities, and induced delirium in stroke patients (12–15). Moreover, physical restraint has negative psychological effects on elderly individuals, such as causing embarrassment, loss of dignity, isolation, anxiety, and other negative emotions (16). Currently, the reduced use of restraint is advocated in different guidelines, and it is stipulated that the use of restraint should be a last resort (17).

With regard to China's elderly care problem, the government has clearly indicated the need to build a policy system and social environment for “support, filial piety, and respect for older persons.” Social concern for the physical and mental health of elderly individuals is gradually increasing. At present, there is a separation of medical and nursing care in China's nursing homes, and combined medical and nursing care services need to be improved (18). Most nursing homes do not have internal wards and cannot provide appropriate medical and health services. Meanwhile, nursing homes in less developed cities do not all have patients sign an informed consent form before performing physical restraint. Therefore, possible complications with physical restraint cannot be dealt with in a timely manner, leading to more serious consequences.

The Chinese Nursing Association officially released a group standard on “Physical Restraint Care for Inpatients” in 2019 (19). This standard was published by the most authoritative nursing organization in China and filled a gap in the guidelines for the physical restraint of inpatients. The group standard defines “minimizing restraint” as the restriction of the free movement of a patient's body part to the smallest extent or for the shortest time. In addition, it defines “restraint alternative” as the use of alternative restraint techniques, such as listening, companionship, the creation of a comfortable and relaxing environment. The group standard follows the principle of “minimizing physical restraint” and requires the active use of restraint alternatives to reduce the use of physical restraint. However, there is no clear regulation on the use of physical restraint in Chinese nursing homes.

Nursing staff are the main participants in the implementation of physical restraint in nursing homes. Studies (20) have pointed out that the implementation and removal of restraint mostly depend on the subjective judgment of nursing staff. Insufficient and incorrect restraint knowledge of nursing staff leads to their negative attitude and improper restraint behavior. The misuse and abuse of restraint violates the decision-making process for minimizing restraint.

Previous studies have shown that the use of physical restraint in nursing homes is influenced by a number of factors, such as activity dysfunction, impaired cognitive status, a high degree of dependence and the prevention of falling in elderly people (9, 10, 21, 22). Among these factors, the most important is to prevent falls (20–22). In addition to the condition of elderly individuals, the attitude and opinion of family members, the knowledge and attitude of nursing staff and the scale of the nursing institution also affect nursing staff's restraint behavior (20, 23). A previous study showed that when nursing staff had only a moderate level of restraint knowledge and attitude, there were still large misunderstandings regarding restraint, and the implementation of restraint did not fully comply with norms. Staff with better knowledge levels and positive attitudes performed better in the implementation of restraint, which suggests that staff need to be further educated (20).

Currently, there is a lack of relevant research on the use of physical restraint in nursing homes in China. In particular, research on physical restraint by nursing staff in elderly care facilities from the perspective of knowledge, attitude, and practice (KAP) is very limited. Kor et al. (17) compared the changes in nursing home staff's KAP regarding the use of physical restraint in Hong Kong but did not explore the influencing factors of KAP. Thus, studying the status quo of the KAP of nursing home staff toward restraint and the influencing factors is urgent to support targeted measures for future restraint training and management, to reduce the use of restraint and avoid adverse consequences, and to provide a reference for the realization of restraint minimization.

The aims of this study were to (1) investigate the current status and explore the influencing factors of nursing staff's KAP regarding physical restraint in elderly care institutions and (2) provide preliminary data support for future training on restraint knowledge to support nursing staff in establishing proper concepts of restraint, optimizing restraint care behavior and standardizing restraint management in elderly care institutions.

## Methods

### Study Design

A cross-sectional survey was performed to explore the status quo of physical restraint and factors affecting nursing staff's KAP regarding restraint.

### Participants

Due to the sensitivity of the research topic, not all nursing homes could actively cooperate with the investigation. Therefore, to ensure the feasibility of the project implementation, the representativeness of the sample and the generalizability of the research results also had to be ensured. Thus, the quota sampling method could be considered a relatively suitable method. This study adopted the method of controlled quota sampling and proportionate allocation according to different dimensions, such as the region and the characteristics of nursing homes. The numbers of institutions drawn from eastern, central, and western Zhejiang Province were 10 (40.0%), 9 (36.0%), and 6 (24.0%), respectively. Among the institutions, 7 were public (28.0%), 10 were private (40.0%), and 8 were public and private (32.0%). The inclusion criteria for nursing homes were as follows: nursing homes with operating qualifications within the scope of Zhejiang Province. Inclusion criteria for nursing staff included (a) having worked in elderly care institutions for at least 1 year and (b) having provided informed consent to participate in the study. The exclusion criteria were nurses or administrators working in care facilities. According to the sample size estimation method for multivariate analysis (24), the sample size needed to be 5–10 times the number of variables. Since 9 variables were expected to be influencing factors in this study, a sample size of 90 was required after taking 10 times the number of variables. Furthermore, the G^*^ Power software package (version 3.1.9.7) was adopted to estimate the sample size (25). In order to obtain a moderate effect size (effect size = 0.15) and sufficient test power (1-β = 0.95, α = 0.05), the recommended minimum sample size is 166. This estimated sample size was increased by 20% to account for any potential missing data, thus yielding an a priori sample size of 208. As such, the study sample of 311 participants clearly satisfied the minimum sample size of 208.

### Data Collection

All data were collected from January to July 2020. The questionnaire consisted of two parts: general information and KAP regarding restraint. The general information questionnaire included questions on gender, age, length of service, education background, title, number of elderly people in charge per day, and restraint training experience. The KAP questionnaire on physical restraint by nurse staff was developed by referring to the Physical Restraints Knowledge, Attitude, and Behavior Scale compiled by Janelli et al. (26) and Scherer et al. (27) as well as the research team's practical work experience.

(a) Knowledge dimension: This dimension included 15 items, including 5 multiple choice questions and 10 single choice questions. The multiple choice questions examined the nursing staff's understanding of restraint tools, applicable conditions, alternative measures, adverse effects and observation points. The single choice questions assessed the nursing staff's understanding of personal rights, informed consent principles, restraint methods, treatment of adverse consequences, legal risks and other contents. The scoring method was as follows: 1 point for correct answers to the multiple choice questions and 0 points for responses that indicated more or fewer answers than the correct answers; 1 point for correct answers and 0 points for wrong answers to the single choice questions. The total score of the dimension ranged from 0 to 15. The higher the score was, the more correct the respondent's restraint knowledge.

(b) Attitude dimension: This dimension contained 11 items that were rated according to a 5-point Likert scale with the options completely agree, agree, uncertain, disagree, and completely disagree, corresponding to 5 points~1 point. The total score of this dimension ranged from 11 to 55 points. Items 1~3 examined the nursing staff's attitudes toward the right to refuse restraint for older persons, their families, and the nursing staff. Item 4 examined nursing staff's attitudes toward older persons and their families' right to know the reasons for restraint. Item 5 assessed the nursing staff's attitudes toward providing psychological support to older persons during restraint. Items 6~9 examined the nursing staff's attitudes toward negative emotions during restraint implementation. Items 10~11 examined the nursing staff's attitudes toward inappropriate reasons for restraint. The higher the score was, the more positive the attitude.

(c) Behavior dimension: This dimension included 17 items, including 1 multiple choice question and 16 single choice questions. The multiple choice question examined the reasons for the realistic use of restraint by nursing staff. One point was awarded for the correct answers to multiple choice answers, and 0 points were for responses that indicated more or fewer answers than the correct answers. Among the 16 single choice questions, 1 question was rated on a 3-point Likert type, corresponding to a score of 1~3; this question examined the restraint decision-making process. The remaining 15 questions were rated on a 4-point Likert type scale, corresponding to a score of 1~4. These items examined the practices of nursing staff in restraint minimization, informed consent, individualized assessment, regular observation and relaxation, psychological support, prevention of pressure ulcers, timely recording, etc.

The higher the score was, the more correct the assessed behavior. Average score rate of each dimension (%) = average score of this dimension/theoretical maximum × 100%.

In the process of developing the questionnaire, 10 experts with rich experience in nursing and scientific research in the geriatric field, such as senior nursing staff and institutional managers, head nurses in the ICU, head nurses in neurology and geriatric departments and nursing teachers in universities, were invited to evaluate the content validity. The item-level content validity index (I-CVI) was >0.80, and the scale-content validity index/universal agreement (S-CVI/UA) was 0.837. Forty subjects were selected for the pilot study. The Cronbach's α coefficients of the three dimensions and the total questionnaire were 0.885, 0.734, 0.933, and 0.910 based on statistical tests, and the test-retest reliability of the questionnaire and each dimension ranged from 0.801 to 0.916. These results showed that the questionnaire had good reliability and validity.

Paper-based questionnaires were used to collect data. Before the survey, the investigators provided uniform guidelines explaining the study purpose and the completion of the survey. The questionnaires were distributed and collected on the spot. During the survey, the investigators were instructed to use neutral, objective, and bias-free language to ask questions and to not reveal their personal views regarding the questions to avoid influencing the respondents to form certain thoughts and provide certain answers. For example, in the assessment of nursing staff's restraint attitudes, one of the items was “Do you think family members of the older person have the right to refuse the use of physical restraint?” Investigators only needed to read the question to the respondents and ask them to give a choice that represented their own opinion from the five options of completely agree, agree, uncertain, disagree, and completely disagree according to their own feelings. It took 15–20 min to complete each questionnaire. A total of 345 questionnaires were distributed, and 15 responses were lost due to the respondent being interrupted by work or being uninterested in the study, with an attrition rate of 4.35%. The remaining 330 staff completed the questionnaire. Among them, 11 respondents with >15% missing responses on the questionnaire and 8 respondents who responded with the same answer, leading the researchers to suspect fraud, were excluded. Finally, 311 valid responses were obtained, for an effective recovery rate of 90.1%.

### Data Analysis

SPSS version 22.0 was used for statistical analysis. The numerical variables were tested for normality, and the results agreed with a normal distribution. Therefore, the mean and standard deviation (SD) were used for statistical description. Categorical variables are represented as frequencies and percentages. Two independent samples *t*-tests or one-way ANOVA were used for comparisons between groups. To explore the relationship between age, length of service and the KAP scores regarding restraint, we transformed age and length of service from numerical variables into categorical variables; that is, age was divided into three levels of ≤ 45 years old, 46–55 years old, and ≥56 years old, and the length of service was divided into three levels of 1~3 years, 4~10 years, and ≥10 years. The basis and criteria for stratification were based on previous literature (28, 29). Pearson correlation analysis was used for bivariate correlations. Multivariate linear regression analysis was used to analyze the influencing factors of nursing staff's physical restraint KAP. *P* < 0.05 was regarded as statistically significant. The analysis showed that the proportions of missing data for numerical data and categorical data were lower than 3.2 and 2.6%, respectively. The missing numerical data were replaced with mean values, and the missing categorical data were replaced with the mode (30).

### Ethics

This study was performed in accordance with the Declaration of Helsinki and approved by the Ethics Committee of the researcher's work unit (Approval No. 2020126). Formal investigation was carried out with the full informed consent of the respondents and the managers of the nursing care institution. We explained the purpose of the survey to the nursing staff in detail; in addition, we promised that the survey would be conducted anonymously and that the data obtained would be strictly managed by the researchers and only used for this study to ensure the privacy of the data obtained. The subjects had the right to withdraw from the study and the right to refuse to answer any particular question(s) at any time.

## Results

### General Characteristics

The age of the nursing staff participating in the survey ranged from 27 to 64 (51.45 ± 7.60) years. A total of 282 (90.7%) were female, and 29 (9.3%) were male. There were 185 (59.5%) with 1–3 years of service, 96 (30.9%) with 4–10 years and 30 (9.6%) with ≥10 years. Among them, 140 (45.0%) had a primary school education or below, 84 (27.0%) had a junior high school education, 65 (20.9%) had a senior high school/technical secondary school education, and 22 (7.1%) had a junior college education/bachelor's degree. A total of 132 (42.4%) had no professional title, 130 (41.8%) were junior workers, and 49 (15.8%) were intermediate workers/senior workers/technicians. There were 235 (75.6%) individuals responsible for ≤ 3 elderly people in full care and 76 (24.4%) responsible for >3. A total of 213 (68.5%) had received restraint training, and 98 (31.5%) had not.

### KAP Status

Among the 311 staff, the score of the knowledge dimension was 7.30 ± 2.07, and the average scoring rate was 48.7%. The attitude dimension score was 41.56 ± 4.81, and the average scoring rate was 75.6%. Among the 140 staff with experience restraining elderly individuals, the practice dimension score was 51.26 ± 10.71, and the average scoring rate was 80.1%. The specific KAP scores are shown in Tables 1–3.

**Table 1:** The scores of physical restraint knowledge (*N* = 311).

**Item**	**Correctness rate** ***n* (%)**
**Below 60% correct**	
What are the adverse effects of restraints?	5 (1.6)
Which of the following do you think are the tools of physical restraint?	10 (3.2)
What should be observed when using restraints on the elderly?	26 (8.4)
Will does physical restraint harm the rights of the elderly?	35 (11.3)
Which of the following do you think are alternatives to physical restraint?	41 (13.2)
In which of the following situations do you think physical restraint should be considered?	51 (16.4)
Does physical restraint for the elderly reduce legal risk for staffs and nursing homes?	62 (19.9)
Do you know how to deal with the adverse effects of physical restraint once they occur?	99 (31.8)
How often are restraints relaxed at least?	140 (45.0)
Should the restraints be close to the old man's body without leaving a gap?	186 (59.8)
**Above 85% correct**	
Before the use of physical restraint, the elderly and their family members should be clearly informed of the reason for restraint and their consent should be obtained.	296 (95.2)
Nursing staff administering restraints to older people must be trained.	286 (92.0)

**Table 2:** The scores of physical restraint attitude (*N* = 311).

**Item**	**Score ($\bar{x}$ ±s)**
**Score ranked the bottom 3**	
I don't think the use of physical restraints saves nursing time or makes staffs less burdened.	2.94 ± 1.22
I don't think physical restraint can be used when staffs are understaffed and unable to care closely for the elderly.	3.23 ± 1.27
I feel embarrassed when family members see me restraining the elderly person.	3.26 ± 1.24
**Score ranked the top 3**	
I think it is important for older people to feel that staffs care about them during restraint.	4.58 ± 0.61
I think it is important for older people and family members to understand the reasons for being restrained.	4.49 ± 0.63
I think that the family members has the right to refuse to use physical restraint on the older person.	4.11 ± 1.01

**Table 3:** The scores of physical restraint practice (*N* = 140).

**Item**	**Correctness rate *n* (%)/score ($\bar{x}$ ±s)**
Accurately grasp the indications of the use of physical restraint.	10 (7.1)
Deciding whether to use physical restraint through collective decision-making.	78 (55.7)
**Score ranked the bottom 3**	
Alternative measures are attempted before restraint.	2.43 ± 1.01
Detailed records are kept of each physical restraint.	2.83 ± 1.23
Cancel the restraint immediately if you feel an older person does not need it.	2.83 ± 0.86
**Score ranked the top 3**	
Respond as soon as possible when the older person becomes irritable, upset or angry during restraint.	3.51 ± 0.72
Regular observation of the older person's mental state and emotional changes during the restraint.	3.45 ± 0.69
Regular checks of blood circulation and skin condition at the restrained area during restraint.	3.41 ± 0.86

### Comparison of Physical Restraint KAP Scores Among Nursing Staff With Different Characteristics

With general information as the independent variable, the differences in the KAP scores of nursing staff with different characteristics were analyzed. First, the results showed that there were statistically significant differences in the scores of the knowledge dimension among nursing staff with different genders, educational backgrounds, professional titles, and training experiences (*P* < 0.05); however, there was no significant difference in restraint knowledge in terms of age, length of service, and number of older persons who required full care (*P* > 0.05). Specifically, men (8.41 ± 1.30) scored higher than women (7.18 ± 2.10) in restraint knowledge. Furthermore, the study showed that the higher the educational level of nursing staff was, the higher the restraint knowledge score. The restraint scores of nurses with a junior high school education, senior high school/technical secondary school education, and junior college education/bachelor's degree were significantly higher than those of nurses with a primary school education or below (*P* < 0.05), but there was no difference between those with a senior high school/technical secondary school education and a junior college education/bachelor's degree (*P* > 0.05). In terms of professional title, the knowledge score of intermediate workers/senior workers/technicians (8.37 ± 1.95) was significantly higher than that of workers without a professional title (7.16 ± 1.53) or junior workers (7.03 ± 2.45), while there was no difference in scores between workers without a title and junior workers (*P* > 0.05). The knowledge score of nursing staff with training experience (7.71 ± 1.90) was higher than that of nursing staff without training experience (6.40 ± 2.16).

Second, the physical restraint attitude scores of nursing staff with different educational backgrounds, professional titles, and training experience were significantly different (*P* < 0.05), while the scores did not significantly differ in terms of age, gender, length of service, and number of older persons who required full care (*P* > 0.05). The restraint attitude score of staff with a junior high school education (43.02 ± 5.46) was significantly higher than that of staff with a primary school or below education (40.48 ± 5.23), while there were no differences between the other groups (*P* > 0.05). In terms of professional title, the results of the LSD *post-hoc* test showed that the attitude scores of intermediate workers/senior workers/technicians were significantly higher than those of staff without professional titles (*P* < 0.05), but there were no differences between the other groups (*P* > 0.05). The attitude score of nursing staff with training experience (42.13 ± 4.17) was higher than that of nursing staff without training experience (40.31 ± 5.81).

Third, the physical restraint practice scores of nursing staff with different lengths of service, education backgrounds, professional titles, and training experiences were significantly different (*P* < 0.05), but the scores did not differ significantly in terms of age, gender, and number of older persons who required full care (*P* > 0.05). Interestingly, the practice scores of staff who had worked for more than 10 years (39.74 ± 8.04) were significantly lower than those of staff who had worked for 1~3 years (53.79 ± 9.18) or 4~10 years (53.00 ± 10.74), while there was no significant difference between the two groups with 1~3 years or 4~10 years of service (*P* > 0.05). The results also showed that the higher the education of the nursing staff was, the higher their practice scores. There were significant differences between the groups with different educational levels (*P* < 0.05), except that there was no difference in the practice score between the junior college/bachelor group and the senior high school/technical secondary school group (*P* > 0.05). Regarding professional title, the restraint practice scores were highest among intermediate workers/senior workers/technicians, followed by junior workers and then workers with not title (*P* < 0.05), while there was no difference between senior workers/technicians and those no professional title (*P* < 0.05). Finally, trained nursing staff scored significantly higher on restraint practice (53.18 ± 10.16) than untrained nurses (40.38 ± 6.52). See Table 4.

**Table 4:** Comparison of KAP scores of physical restraint among nursing staff with different characteristics (score, $\bar{x}$ ± s).

	**Item**	**Knowledge dimension** **(*n* = 311)**	**Attitude dimension** **(*n* = 311)**	**Practice dimension** **(*n* = 140)**
Age (years old)	≤ 45	7.86 ± 2.33	42.34 ± 3.84	52.93 ± 11.38
	46~55	7.21 ± 1.96	41.16 ± 5.10	50.79 ± 10.48
	≥56	7.09 ± 2.05	41.77 ± 4.83	50.00 ± 10.27
*F*		2.796	1.421	0.865
*P*		0.063	0.243	0.423
Gender	Male	8.41 ± 1.30	42.86 ± 1.30	54.50 ± 9.39
	Female	7.18 ± 2.10	41.42 ± 5.02	50.53 ± 10.90
*t*		3.096	1.537	1.718
*P*		**0.002**	0.125	0.088
Length of service (year)	1~3	7.51 ± 1.87	41.48 ± 3.83	53.79 ± 9.18
	4~10	6.96 ± 2.09	41.98 ± 5.56	53.00 ± 10.74
	≥10	7.03 ± 2.93	40.70 ± 7.21	39.74 ± 8.04
*F*		2.565	0.870	20.474
*P*		0.079	0.420	**<0.001**
Educational background	Primary school or below	6.28 ± 1.82	40.48 ± 5.23	44.74 ± 8.68
	Junior high school	7.21 ± 1.99	43.02 ± 5.46	49.14 ± 10.68
	Senior high school/technical secondary school	8.92 ± 1.28	41.86 ± 2.72	56.47 ± 9.49
	Junior college/Bachelor	9.27 ± 1.24	41.91 ± 2.07	59.85 ± 5.08
*F*		44.630	5.275	15.306
*P*		**<0.001**	**0.001**	**<0.001**
Professional title	No title	7.16 ± 1.53	40.77 ± 3.60	53.63 ± 9.38
	Junior workers	7.03 ± 2.45	41.88 ± 5.69	49.33 ± 11.06
	Intermediate workers/Senior workers/Technician	8.37 ± 1.95	42.82 ± 4.86	54.16 ± 10.40
*F*		8.288	3.781	3.172
*P*		**<0.001**	**0.024**	**0.045**
Number of older persons	≤ 3	7.34 ± 2.14	41.70 ± 4.37	51.59 ± 11.03
required full caring	>3	7.16 ± 1.86	41.11 ± 6.01	49.85 ± 9.26
*t*		0.668	0.939	0.747
*P*		0.505	0.348	0.457
Trained experience	No	6.40 ± 2.16	40.31 ± 5.81	40.38 ± 6.52
	Yes	7.71 ± 1.90	42.13 ± 4.17	53.18 ± 10.16
*t*		−5.420	−2.797	−5.567
*P*		**<0.001**	**0.006**	**<0.001**

*The bold values indicate P values less than 0.05, which are statistically significant*.

### Correlation Analysis of KAP Scores

The results showed that there was a positive correlation between restraint knowledge and attitude (*r* = 0.265), knowledge and practice (*r* = 0.699), and attitude and practice (*r* = 0.314) among nursing staff who had implemented restraint (*P* < 0.05).

### Multiple Linear Regression Analysis on Influencing Factors of Nursing Staff's KAP Regarding Physical Restraint

The physical restraint KAP scores of nursing staff were taken as dependent variables, and the variables with statistical significance (*P* < 0.05) in univariate analysis and other variables considered meaningful in terms of practical experience were included for multiple linear stepwise regression analysis. Categorical variables such as educational background, professional title, and length of service were converted into dummy variables and included in multiple linear regression analysis (α in = 0.05, α out = 0.10). Multivariate analysis of the behavior dimensions added knowledge and attitude dimension scores as independent variables apart from the demographic data.

The results showed that education background and training experience were protective factors for the knowledge scores of nursing staff. Staff with a junior high school, senior high school/technical secondary school, or junior college/bachelor education scored significantly higher than those with a primary school or below education (β = 0.178, *P* = 0.001; β = 0.452, *P* = 0.000; β = 0.304, *P* = 0.000). The knowledge scores of staff with training experience were significantly higher than those of staff without training (β = 0.129, *P* = 0.015).

Additionally, the results demonstrated that training experience, educational background and professional title were significant predictors of attitude. The attitude scores of staff with training experience were significantly higher than those of staff without training (β = 0.200, *P* = 0.001). There were statistically significant differences in the attitude scores between those with a junior high school education and those with a primary school or below education (β = 0.199, *P* = 0.001). Furthermore, the attitude scores of junior workers and intermediate workers/senior workers/technicians were significantly higher than those of staff without professional titles (β = 0.163, *P* = 0.007; β = 0.195, *P* = 0.002).

Finally, restraint knowledge and length of service were the factors significantly associated with practice scores. Specifically, the higher the score of restraint knowledge was, the more correct the practice. The practice scores of those with more than 10 years of service were significantly lower than those with 1~3 years of service (β = −0.224, *P* = 0.003). See Table 5.

**Table 5:** Multiple linear regression analysis on influencing factors of physical restraint knowledge, attitude, and practice of nursing staff.

**The dependent variable**	**The independent variables**	***B***	**Standard error**	**β**	***t***	***P***
Knowledge score^†^	Constant	6.216	0.747		8.322	<0.001
	**Educational background**					
	Primary school or below (ref)					
	Junior high school	0.827	0.256	0.178	3.229	0.001
	Senior high school/technical secondary school	2.297	0.295	0.452	7.781	<0.001
	Junior college/	2.451	0.433	0.304	5.659	<0.001
	**Trained experience**	0.574	0.234	0.129	2.450	0.015
Attitude score^‡^	Constant	38.464	0.624		61.623	<0.001
	**Trained experience**	2.068	0.620	0.200	3.337	0.001
	**Educational background**					
	Primary school or below (ref)					
	Junior high school	2.159	0.649	0.199	3.329	0.001
	Senior high school/technical secondary school	0.009	0.766	0.001	0.012	0.990
	Junior college/Bachelor	0.301	1.140	0.016	0.264	0.792
	**Professional title**					
	No title (ref)					
	Junior workers	1.589	0.589	0.163	2.698	0.007
	Intermediate workers/Senior workers/Technician	2.573	0.834	0.195	3.086	0.002
Practice score^§^	Constant	23.013	4.132		5.569	<0.001
	**Knowledge score**	3.014	0.452	0.576	6.665	<0.001
	**Length of service (year)**					
	1~3 (ref)					
	4~10	1.805	1.500	0.076	1.204	0.231
	≥10	−6.458	2.114	−0.224	−3.056	0.003

† *R^2^ = 0.330, adjusted R^2^ = 0.310, F = 16.468, P < 0.001*.

‡ *R^2^ = 0.104, adjusted R^2^ = 0.086, F = 5.878, P < 0.001*.

§ *R^2^ = 0.590, adjusted R^2^ = 0.562, F = 20.781, P < 0.001*.

## Discussion

### The Level of Restraint Knowledge of Nursing Staff Is Generally Low and Needs to Be Improved

The results of this survey showed that nursing staff's knowledge level regarding restraint was relatively low (the average scoring rate was only 48.7%), which may be due to the lower education levels of respondents in this study (only 7.1% of nursing staff had higher education). The second possible reason is that 31.5% of respondents did not receive restraint training. In addition, the low level of knowledge may be related to the late start of the standardized management of restraint in China and the fact that less attention has been given to the restraint of elderly individuals by pension institution practitioners. Although 68.5% of staff had received some restraint training, the results in Table 1 suggested that the knowledge acquired by staff was still one-sided or somewhat misleading, indicating that the previous restraint training may not have been systematic and comprehensive.

For example, 306 (98.4%) of the staff lacked awareness of the harmful effects of using physical restraint, which may affect the correctness of their decisions to use restraint. A total of 301 (96.8%) staff members had an incorrect understanding of restraint instruments, so they tended to misunderstand the concept of restraint and to view restraint as a protective measure to prevent elderly injury, which is one of the reasons that affects restraint minimization (31). A total of 276 (88.7%) of the staff believed that physical restraint would not violate the personal rights of elderly individuals, which indicates that the staff have a substantial misunderstanding on this point, consistent with the research of Eskandari et al. (20). In fact, physical restraint is considered to violate the autonomy, freedom and risk-taking rights of the restrained person, limit their right to activities and generate negative emotions such as frustration, fear, and depression (9, 14). From an ethical perspective, nursing staff should try their best to protect the dignity and quality of life of elderly individuals when using restraint; however, nursing staff often ignore the rights of elderly individuals in the process of using restraint (9). It is therefore necessary to guide caregivers in developing the correct perception that the views of older people themselves and their families must be fully considered before restraint is applied. A total of 86.8% of staff could not correctly identify alternative measures to restraint. Studies have pointed out that restraint should be regarded as the last resort; that is, only when all alternative measures are ineffective and the potential benefits of using physical restraint outweigh the potential harm can restraint be used to protect the safety of elderly individuals (13). A summary of evidence on alternative restraint measures recommended the following measures as Level A evidence (32): comprehensive assessment of patient needs (illness, psychological, physical), multidisciplinary teamwork, relaxation therapies such as musical acupuncture and massage, removal of the catheter from the patient's view, timely, and dynamic assessment of the patient's physical changes, avoidance of falls and bed fall injuries, and provision of a quiet and comfortable ward environment. These measures can be used to avoid restraint by changing the surrounding environment or providing subjective support. In practical work scenarios, distraction, the provision of a familiar family-like environment, physical touch, and increased communication and other means can be used as alternative measures (31). Only when staff are aware of the concept and application of alternative restraint measures can it be more helpful to minimize restraint. In summary, in terms of the score of the knowledge dimension, the nursing staff's knowledge of restraint is still not comprehensive enough. This indicates that it is essential and urgent to further strengthen the training frequency, enrich the training content, and encourage nursing staff to build a complete restraint knowledge system to guide nursing practice in the future. According to the results of this study, systematic training on restraint can be considered to strengthen knowledge on alternative measures of restraint, the applicable conditions of restraint, specific methods of restraint, adverse effects, and treatment measures. Curriculum design can be considered in two forms: classroom-based and network-based. In addition to teaching basic knowledge, classroom-based courses can be considered to encourage nursing staff to share past experiences of restraint. Web-based courses ensure that nursing staff can learn anytime, anywhere. The combination of the two forms of curriculum design can meet the different requirements of learning.

### The Restraint Attitude of Nursing Staff Is Above a Moderate Level in Terms of Correctness but Still Needs Improvement

The results showed that the staff generally had positive attitudes toward restraint, but they had negative or improper attitudes for some items. For example, the item “I do not believe that the use of physical restraint saves nursing time or makes the staff less burdened” had the lowest score. Staff misunderstanding of the purpose of physical restraint makes it possible for them to use restraint for the purpose of avoiding or reducing the burden of care. This is contrary to standards of ethics and morality and will lead to misuse and abuse of restraint, which will inevitably increase the incidence of adverse outcomes caused by restraint. Furthermore, the item “I do not believe that physical restraint can be used when staff are understaffed and unable to care closely for elderly individuals” had the second-to-lowest score. A study in China showed that the low nurse–patient ratio and the heavy workload of nurse staff constitute one of the reasons for the high use rate of physical restraint (13). At present, there is an even more serious shortage of nursing staff in most nursing homes (33). This misunderstanding of restraint may lead nursing staff to choose the use of restraint to reduce their work when they are busy. For this purpose, restraint is relatively simple in procedures for which evaluation, recording, and observation are usually omitted, which not only fail to achieve the desired effect but are more likely to cause adverse effects. In fact, the implementation of restraint following established norms often takes more time for staff (34). In addition, the item “I feel embarrassed when family members see me restraining the elderly person” had the third-lowest score. A possible reason for the nursing staff's feeling of embarrassment is that they are taking actions that they believe are inappropriate. Although nursing staff think that restraint may hurt the older person, they still use it to avoid responsibility for care. In other words, only when elderly care workers always hold a prudent, respectful and responsible attitude toward restraint can the implementation of restraint be more in line with a humanistic ethical spirit. Therefore, it is worth emphasizing that appropriate constraint decision-making must be based on the correct values and moral judgment.

### There Is Still Room for Further Improvement in the Restraint Behavior of Nursing Staff

In terms of restraining practice, first, the lowest-scoring item was “Alternative measures are attempted before restraint.” Studies have found that the majority of staff support the use of physical restraint rather than prioritizing alternative measures in regard to preventing unexpected situations in elderly individuals (35). Jia et al. (31) also pointed out that lack of knowledge and support for alternative measures of restraint is one of the factors affecting the minimization of physical restraint. In this study, the main reason for the nursing staff's non-standard restraint decisions was the lack of knowledge of alternative restraint measures. Moreover, there was a general lack of records of physical restraint among nursing staff in this study, which was consistent with the findings of Almomani et al. (36). Effective and complete records of restraint are helpful for nursing staff to grasp the overall situation of elderly restraint, identify, and deal with the adverse effects of restraint in time, and provide a reference for when to terminate restraint. Therefore, it is imperative to include a teaching component on restraint recording in future training. The item “Terminate restraint immediately if you feel an older person does not need it” had the third-lowest score. For elderly individuals in stable condition, if nursing staff does not terminate restraint in time, the risk of falling and pressure ulcers may be increased, and the elderly individual may experience negative emotions. In addition, the results showed that only a small proportion (7.1%) of staff accurately grasped the indications for the use of restraint, which easily led to the misuse and abuse of restraint, resulting in subsequent negative effects. These studies also revealed that only 55.7% of staff often made restraint decisions through collective decision-making in routine care. The restraint decision-making process is easily affected by the subjective knowledge, attitude, and intention of caregivers (20). To reduce the unreasonable use of restraint, many factors need to be considered in the decision-making process, including the results of expert consultation, relevant normative guidance, cooperation of various personnel (nursing staff, family members, patients), and the priority use of alternative measures (37).

### Nursing Staff's KAP Regarding Restraint Were Affected by Many Factors

This study further explored the influencing factors of the physical restraint KAP of nursing staff. The results showed that educational background was the influencing factor of physical restraint knowledge and attitude; that is, nursing staff with lower academic qualifications had poorer knowledge and more negative attitudes regarding restraint use. This finding was basically consistent with other previous studies (36, 38). However, the nurses in this study had lower educational backgrounds than those included in the above two studies. The possible reasons for the significant association of educational background with knowledge and attitude might be that staff with higher education have more opportunities to learn specialized knowledge and tend to have higher learning ability (39) so that they can actively pay attention and effectively acquire relevant knowledge of physical restraint. The results indicate that improving the academic qualifications of nursing staff in the future to meet the needs of more specialized care is an urgent task.

Professional title had a positive effect on restraint attitude. This might be because nursing staff with higher professional titles usually have rich life experience and vocational training experience and are more objective and accurate in grasping the legal and ethical boundaries of nursing behavior (29, 40).

This finding also indicated that the training experience of nursing staff was an influencing factor not only of knowledge but also attitude toward restraint, which was consistent with other previous similar studies (20, 38). A possible reason might be that training is a process of transforming information and skills for trainees to have better awareness and attitudes (20). Thus, training plays an extremely important role in correcting incorrect viewpoints regarding restraint and establishing correct professional ethics, and it is imperative to carry out effective training on physical restraint.

Our study demonstrated that restraint knowledge and length of service were influencing factors of practice. When implementing restraint, staff with comprehensive knowledge will be more able to consider the possible effects of restraint from the perspectives of the older person's safety, mental health, and restraint ethics, and their behavior is usually more correct and standardized (41, 42). This is consistent with previous research results (43).

In this study, it was also found that the longer the length of service, the lower the score for restraint practice. As seen from the results of univariate analysis, nursing staff with more than 10 years of working experience had the lowest practice scores. The reason may be that most of the staff with more working years have a lower educational background in China. A large portion of these nursing staff are middle-aged persons from less developed rural areas. They have formed poor behavioral habits and solidified wrong ways of thinking in their long-term working environment (44).

The practices of nursing staff are also influenced by the lack of training related to the physical restraint for caregivers in Chinese elderly institutions and the cultural environment of institutional care safety (7). Even though some training has been carried out in recent years in China, there is still no significant effect for nursing staff with a longer length of service. Thus, it can be seen that staff with long working experience (especially over 10 years) and a low level of restraint knowledge are the key targets for future restraint training, and their knowledge and skills need to be continuously reinforced.

## Limitations of the Study and Future Recommendations

There are some limitations to this study. First, the sample was from one province of China, and a non-random sampling method was used, which limits the generalizability of our findings. Thus, multicenter research conducted in several provinces across the country is necessary in the future. Second, restraint is a relatively sensitive topic, and although it was fully explained to the nursing staff before the survey that their personal privacy would be protected, some of them may still not have completed the questionnaire objectively and may have deliberately obscured the real situation, which may have led to information bias in the results. Third, because of the large gender gap in the survey, more research is needed to confirm whether gender is an influencing factor of restraint KAP. Fourth, the study was cross-sectional and only captured a snapshot of the practices being evaluated at a specific time. The data obtained are only indicative of the current situation and cannot be used to make judgments about cause and effect. Therefore, longitudinal studies can be used to analyze the influencing factors more precisely in the future. Finally, this study did not qualitatively explore in depth the reasons for the KAP status quo; thus, a qualitative method should be used for further study.

## Conclusions

Nursing staff have a low level of knowledge about restraint, and their attitude toward restraint is not completely positive. There are also weaknesses in the specific practice of restraint. Government departments and managers of pension institutions need to pay attention to physical restraint training and education among nursing staff, focus on correcting their wrong ideas and opinions and changing their improper behaviors related to restraint to improve the norms regarding the use of physical restraint.

## Data Availability Statement

The datasets presented in this article are not readily available because the datasets generated for this study will not be made publicly available for ethical reasons. We do not have the permission of the organizations involved in the research. Requests to access the datasets should be directed to the datasets generated for this study will not be made publicly available for ethical reasons.

## Ethics Statement

The studies involving human participants were reviewed and approved by Medical Ethics Committee of Wenzhou Medical University (Approval No. 2020126). The patients/participants provided their written informed consent to participate in this study.

## Author Contributions

YL, YW, JL, and HX made substantial contributions to conception and design, data analysis, and interpretation. YL, YG, YW, DG, SJ, and HX involved in data collection, drafting the manuscript or revising it critically for important intellectual content. YL, YG, YW, DG, SJ, JL, and HX given final approval of the version to be published. Each author should have participated sufficiently in the work to take public responsibility for appropriate portions of the content and agreed to be accountable for all aspects of the work in ensuring that questions related to the accuracy or integrity of any part of the work are appropriately investigated and resolved. All authors contributed to the article and approved the submitted version.

## Funding

This research was supported by the Natural Science Foundation of Zhejiang Province (LGF20H250003), Wenzhou Science and Technology Bureau (Y20190157), and the National Undergraduate Innovation and Entrepreneurship Training Program (201910343038).

## Conflict of Interest

The authors declare that the research was conducted in the absence of any commercial or financial relationships that could be construed as a potential conflict of interest.

## Publisher's Note

All claims expressed in this article are solely those of the authors and do not necessarily represent those of their affiliated organizations, or those of the publisher, the editors and the reviewers. Any product that may be evaluated in this article, or claim that may be made by its manufacturer, is not guaranteed or endorsed by the publisher.
